# Re-considering the Role of Sleep Hygiene Behaviours in Sleep: Associations Between Sleep Hygiene, Perceptions and Sleep

**DOI:** 10.1007/s12529-023-10212-y

**Published:** 2023-09-06

**Authors:** Thomas McAlpine, Barbara Mullan, Patrick J. F. Clarke

**Affiliations:** 1 enAble Institute, Curtin University, Bentley, WA 6102 Australia; 2 School of Population Health, Curtin University, Bentley, WA 6102 Australia

**Keywords:** Sleep hygiene, Sleep, Perseverative cognition, Routine, Negative emotionality, Perceptions

## Abstract

**Background:**

Sleep hygiene behaviours are a suggested set of behaviours people can engage in to improve sleep. However, there are numerous issues relating to the measurement of sleep hygiene, primarily, the lack of consensus as to which behaviours impact sleep and should therefore be included in scales.

**Method:**

Cross-sectional correlational methods were used to assess the association between sleep quality, a highly inclusive range of sleep hygiene behaviours, and individual perceptions of those behaviours in a non-clinical sample of 300 participants.

**Results:**

Of the 35 sleep hygiene behaviours assessed, 18 were independently associated with sleep quality. Post-hoc factor analysis revealed that behaviours clustered together across four factors. A ‘routine’ factor included behaviours such as going to bed and waking up at the same time each night, and were important predictors of sleep quality, as were behaviours belonging to the ‘perseverative cognition’ and ‘negative emotionality’ factor. Other behaviours related to physiological processes like exposure to sunlight during the day and going to bed hungry were also significantly associated with sleep. Negative perceptions moderated the relationship between daytime exposure to sunlight and sleep.

**Conclusions:**

Although certain behaviours were significantly related to sleep, almost half were not, supporting the need to examine the association between sleep and behaviours used for sleep hygiene recommendations more critically. Reframing sleep hygiene recommendations into a condensed set of shared underlying mechanisms may be of benefit for the development of sleep hygiene scales and interventions in non-clinical populations.

**Supplementary Information:**

The online version contains supplementary material available at 10.1007/s12529-023-10212-y.

## Introduction

Insufficient sleep is estimated to effect between 20 and 35% of adults at any given time [1, 2]. Aside from the associated short-term repercussions on daytime functioning like fatigue and decreased cognitive performance [3, 4], consistent sleep deficiency is also related to other adverse health outcomes such as psychological disorders, cardiovascular disease, and obesity [5, 6]. In addition to the considerable adverse consequences for the individual, these negative outcomes combine to place a large burden on the economy. For example, estimates of the impact of insufficient sleep on the economies of developed countries range from 1.35% to nearly 3% of annual Gross Domestic Product. In the United States, this equates to $411 billion per year, and includes the costs of increased mortality risk, but also indirect costs such as workplace and motor vehicle accidents due to sleep deficiency, and the loss of productivity in the workplace [1, 7]. Importantly, the sleep problems that contribute to these estimates are experienced by a roughly even combination of non-clinical populations and populations with specific sleep disorders [1], which highlights the need to also examine the mechanisms that drive poor sleep in non-clinical populations.

Given the importance of sleep for health and everyday functioning, considerable research effort has been devoted to identifying these mechanisms. One example has been a focus on sleep hygiene behaviours, which are modifiable behaviours that can be beneficial or detrimental to sleep [8]. The promotion of sleep hygiene behaviours is typically targeted at the general population with the goal of improving sleep quality. However, scales that measure sleep hygiene are specifically based off criteria set forth in the International Classification of Sleep Disorders [9, 10] and much of our understanding of these behaviours is derived from studies involving clinical samples [10, 11]. While these populations offer valuable insights into extreme cases of sleep disorders and specific sleep hygiene behaviours contributing to these pathologies, their applicability to the general population may be limited. This is due to the broader variability in sleep habits found in the general population compared to those with clinical disorders [12]. Therefore, examination of the associations between sleep hygiene behaviours and indicators of sleep quality in the general population is critical to inform the development of more effective public health strategies and interventions for promoting sleep hygiene practices. However, there are several further issues with the conceptualisation of sleep hygiene behaviour and its measurement which can impede the advancement of research in this field.

**Fig. 1:**
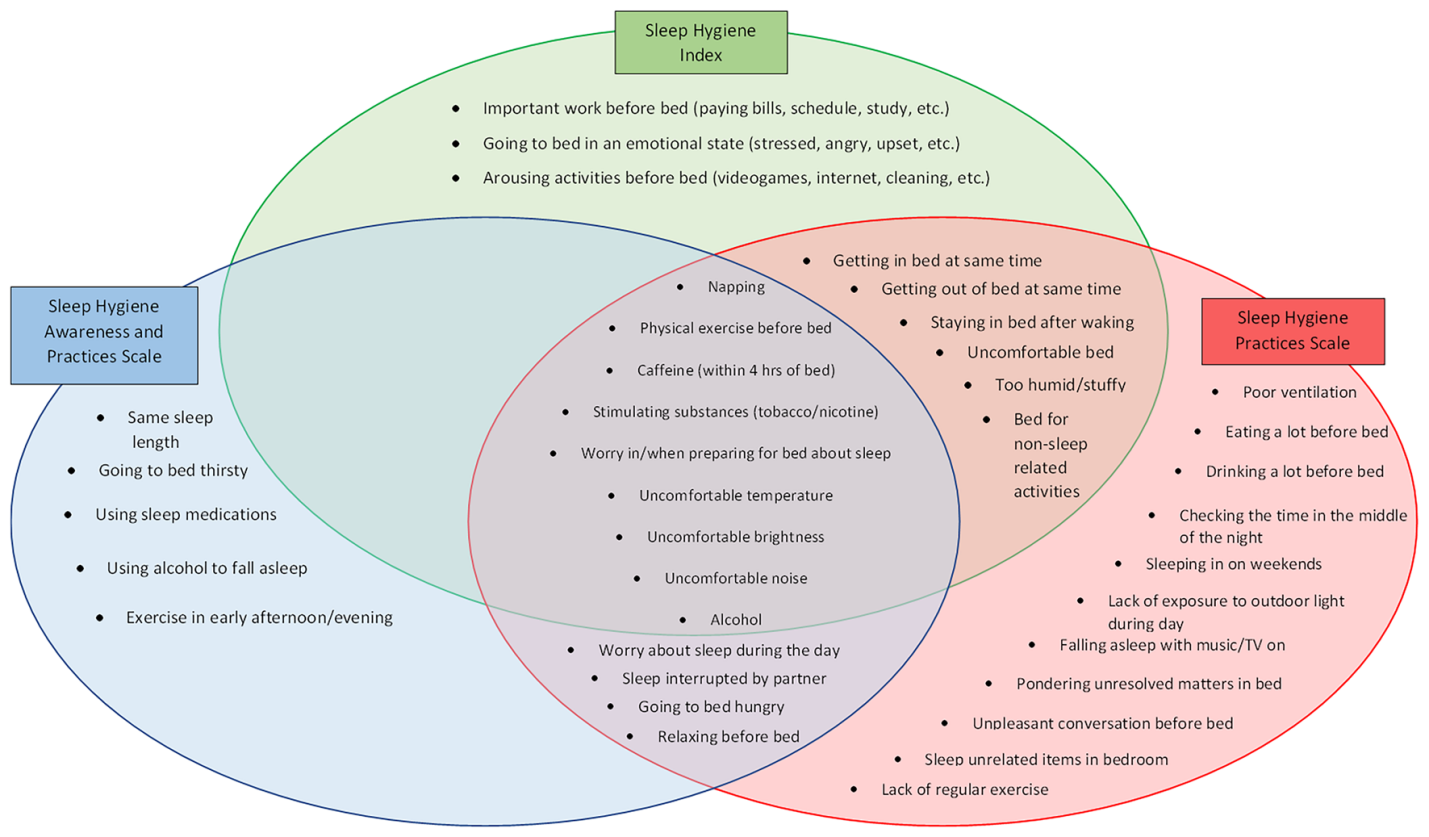
Sleep hygiene behaviour overlap across scales

One major issue surrounding sleep hygiene measurement is that surprisingly little consensus exists on which behaviours are the most important for maintaining healthy sleep patterns. Sleep hygiene scales differ substantially on the particular behaviours assessed underscoring inconsistency regarding which behaviours are integral to better sleep outcomes [13]. For example, the most common scales, the Sleep Hygiene Index [14] and the Sleep Hygiene Awareness and Practices Scale [15] assess 13 and 19 separate sleep hygiene behaviours respectively, while other scales like the Sleep Hygiene Practices Scale [16] assess 30. While some overlap exists in behaviours between scales, many are unique to each scale. In addition, many behaviours, such as screen use, have emerged more recently and are not included in many of them (see Fig. 1 for a depiction of the commonalities and differences between scales).

A separate measurement issue pertains to the representation of seemingly similar behaviours across multiple items within scales. For example, the Sleep Hygiene Index [14], lists doing important work before bed (e.g., paying bills, studying) separately from engaging in arousing activities before bed (e.g., playing video games, cleaning the house). The distinction between these two items is not particularly clear, especially to participants who may be completing these questionnaires, since it can be argued that doing important work before bed could include arousing activities. This confusion is compounded by the lack of clarity surrounding ‘arousal’, since arousing activities could be cognitive and/or physiological. To elaborate, it is possible that these items are affecting sleep by the same mechanism (i.e., doing important work implies an elevated level of cognitive arousal which may also be assessed by the other item). Nevertheless, they are treated as independent.

The lack of consensus as to which behaviours are linked to better/worse sleep is further confounded by the inclusion of behaviours that are not clearly associated with sleep disturbance in non-clinical populations. For example, one of the most longstanding and commonly used sleep hygiene measures [14, 16] asks about the use of bed for activities other than sleep and sex. The use of the bed for non-sleep related behaviours (e.g., eating, reading, watching television) is suggested to decrease the association of the bed with sleep and consequently decrease the ease with which individuals are able to fall asleep [17]. While intuitively appealing, this association has not been demonstrated for many common non-sleep related behaviours performed in bed such as reading, watching TV, lounging, and talking on the phone [18]. It is critically important to establish which sleep hygiene behaviours are of intrinsic value from a public health perspective. Therefore, a more holistic understanding of these inconsistent associations is warranted.

One possible factor contributing to these inconsistencies is the role of individual perceptions/beliefs about sleep hygiene. Research has demonstrated that both knowledge and beliefs about sleep hygiene behaviours, as assessed using sleep hygiene knowledge or belief questionnaires, vary substantially between populations, evidenced by different total scores and large standard deviations (e.g., [19, 20]), underscoring different beliefs about which behaviours are important for an individual’s sleep. Research has demonstrated that rigid and/or negative or unhelpful beliefs about *sleep* (e.g. “I must get 8 h of sleep to feel refreshed and function well the next day”) are more prevalent for those with insomnia than those without [21]. This may be due to such negative beliefs exacerbating worry about sleep-related requirements, which can include an exaggerated focus on potentially faulty sleep-promoting practices, leading to heightened emotional arousal and the consequent impairment of sleep [22]. Therefore, unhelpful, or negative *perceptions* about sleep hygiene behaviours may similarly influence sleep (e.g., "I believe that going to bed at different times each night impairs my sleep"), however no study to date has examined this association. Given the suggested mechanism via which negative beliefs influence sleep, more negative perceptions of sleep hygiene behaviours may worsen the relationship between frequency of performance and sleep quality (i.e., by heightening arousal levels due to an exaggerated focus on performance).

Thus, the aim of this research was twofold. Firstly, we aimed to assess and compare the association of a highly inclusive range of sleep hygiene behaviours with sleep outcomes as an initial attempt to address some of the issues raised and establish which of the currently used sleep hygiene behaviours are related to sleep in a non-clinical population. Secondly, we aimed to investigate whether perceptions of sleep hygiene were related to sleep after accounting for performance of sleep hygiene behaviours and could therefore be considered responsible for some of the inconsistencies previously discussed. To address these aims, a cross-sectional design was used, where participants were asked about their behaviours over the previous week and were given a self-report measure of sleep quality. They were also asked about their perceptions of the impact of each sleep hygiene behaviour on their sleep. Given the numerous sleep hygiene behaviours examined and the contradictory nature of the existing research for some, no hypotheses are offered for the association of each sleep hygiene behaviour on sleep. However, we hypothesised that perceptions of sleep hygiene behaviours would be related to sleep, after controlling for behavioural performance. Finally, we hypothesised that for any association of behaviour with sleep, the relationship would be exaggerated for those with more negative perceptions when compared to those with more favourable perceptions.

## Method

### Participants

Participants were recruited using the online paid participant pool Prolific, as well as by advertising through social media from November 2020 to January of 2021. A second wave of participants were recruited in January 2023. In both waves, participants were sampled by quota to ensure an approximately even number of good and poor sleepers. A total of 343 participants were recruited, however 22 participants did not complete at least 50% of the questionnaire, 20 participants were not invited to complete the main questionnaire due to quota restrictions, and 1 participant was excluded due to failing both attention check items. This left an approximately even number of participants from each wave (Wave 1 = 160, Wave 2 = 140) to equal 300 participants in the final sample.

Participants in Australia were offered the chance to enter a prize draw for one of five $50 retail vouchers, while participants in the UK were paid £2.23 (approximately minimum wage) for their participation. The 20 participants who were excluded due to quota restrictions were reimbursed £0.23 for completing the pre-screen.

### Measures

#### Sleep Quality

Sleep Quality was measured using the Pittsburgh Sleep Quality Index (PSQI). The PSQI is a validated scale that contains seven components assessing different aspects of sleep over the previous month such as subjective sleep quality, sleep duration, and sleep efficiency. The scores from the components are summed to create an overall score ranging from 0 to 21. Higher scores indicate poorer sleep quality. For the purpose of quota sampling, those that scored > 5 were considered poor sleepers, and those that scored ≤ 5 were considered good sleepers, consistent with previously used cut-offs [23]. Items were adapted to ask about sleep over the previous week, to align with items from the sleep hygiene component of the survey.

#### Sleep Hygiene Behaviours

Sleep Hygiene was measured by pooling items across three different sleep hygiene scales: The Sleep Hygiene Index [14], the Sleep Hygiene Practices Scale [16], and the practices component from the Sleep Hygiene Awareness and Practices Scale [15]. Items unique to each scale were retained in their existing form, but similar items were adapted to best capture the fundamental meaning of both. For example, “Do you worry as you prepare for bed about your inability to sleep at night?” from the Sleep Hygiene Awareness and Practices Scale, and “Do you worry about not being able to fall asleep in bed” from the Sleep Hygiene Practices Scale, were converted to “Have you worried about falling asleep while in bed?”. For discrepancies between scales that involved time-based differences (e.g., exercising within 1h of bed, exercising within 2h of bed), the item was combined and then adapted to allow the participant to specify the time before bed that the sleep hygiene behaviour was performed. Several more items were also added from recent research highlighting potential influences on sleep for behaviours not listed in any of the three sleep hygiene scales (e.g., exposure to blue light before bed, having sleep interrupted by pets).

The resultant measure contained 35 items, each of which followed the same stem question: “Have you…”. Participants responded on a 4-point Likert scale by rating the frequency that they had engaged in the item over the previous week. Responses ranged from “Not during the past week” to “5 or more times in the past week”.

#### Sleep Hygiene Perceptions

To capture participants’ perceptions of how each item affected their sleep, perceptions of the impact of each of the 35 behaviours were also assessed. An anchor statement was provided ("Please select the option that best describes how much you believe each of the following behaviours affect your sleep") and participants responded on a 7-point Likert scale by rating the degree to which they believed that each item impaired or improved their sleep, with specific instructions to consider how it would affect them personally (not how it might affect everyone else generally). Responses ranged from “Strongly impairs my sleep” to “Strongly improves my sleep”, with “Doesn’t affect my sleep” as the middle option.

#### Sociodemographic and Control Variables

Participants were asked to report their age, gender, ethnicity, and highest level of education. Education levels were aggregated across UK and Australian participants so that the variable could be analysed similarly across both samples. Two control questions were also asked; “Do you have any children aged 2 years or younger living in your household?”, and “Have you ever been diagnosed with a sleep disorder?”. These questions were included to control for the effect of known covariates [6, 24] of sleep quality that were not the primary focus of the study.

### Statistical Analysis

Data were screened for errors and missing values. Expectation maximization was used to impute missing data for continuous variables. Demographic and control variables were assessed for their relationship to the outcome variable. Independent samples t-tests revealed a significant difference in PSQI scores between those who reported a previous diagnosis of sleep disorder and those who did not (*p* = .018), as well as the country participants were recruited from *(p* = .039)*,* so both were included as control variables. No other demographic or control variables were found to be related to sleep. All analyses were conducted using IBM SPSS Statistics for Windows, Version 28.

#### Sleep Hygiene Behaviours

To examine the ability of each behaviour under consideration to independently account for variance in sleep quality, bivariate correlations were initially used to assess the relationship with the outcome variable (Supplementary 1). The 18 variables that significantly correlated were included for analyses using multiple regression so that comparisons of the associations between variables and sleep could be made using standardised regression coefficients. Multicollinearity as assessed using the Variance Inflation Factor (VIF) was deemed not to be problematic as values were all below two [25]. Other assumptions for multiple regression analyses were assessed and only minor violations occurred^1^.

#### Sleep Hygiene Perceptions

To assess our hypotheses relating to sleep hygiene perceptions hierarchical multiple regression analyses were conducted to predict PSQI scores. Country of origin and previous diagnosis of sleep disorder were added in the first block, sleep hygiene behaviours were added in the second block, sleep hygiene perceptions were added in the third block, and the interaction term in the final block. Continuous predictors were standardised in line with recommendations for assessing interaction terms using regression [26]. These analyses were conducted individually for each of the 18 behaviours that were correlated with sleep and were assessed against Bonferroni corrected significance levels (*p* < .003) instead of a single regression including all behaviours at once. This was so the interaction effects could be isolated and interpreted properly. To interpret significant interactions, simple slopes analyses were conducted using practically relevant thresholds for perception groups. Specifically, scores that indicated negative perceptions ("Strongly/Moderately/Slightly impairs my sleep") were compared to scores that represented neutral or positive perceptions ("Does not affect my sleep" or "Strongly/Moderately/Slightly improves my sleep") and the relationship between sleep hygiene performance and sleep was assessed at each level.

#### Post-hoc Analyses

Given the bivariate associations between some of the sleep hygiene behaviours, and the potential overlap in content (e.g., waking up at different times, going to bed at different times), a factor analysis was conducted post-hoc to explore the potential factor structure of the significant sleep hygiene behaviours.

## Results

Categorical demographic and control variables are summarised in Table 1. Previous diagnosis of sleep disorder and country of origin were related to sleep, whereby those with a previous diagnosis reported PSQI scores that were 2.24 points higher on average than those without a previous diagnosis, 95% CI [0.39, 4.09], *t*(294) = 2.38, *p* = .018, and UK participants reported PSQI scores that were 0.86 points higher on average than Australian participants, 95% CI [0.05, 1.67], *t*(298) = 2.08, *p* = .039.The mean age of the total sample was 34.7 (SD = 11.7) and was not significantly related to PSQI scores (*p* = .600) nor were the remaining demographic or control variables.

**Table 1:** Summary of demographic characteristics overall and by sleep category

	Overall	Sleep Quality	
Gender	% (n)	Poor % (n)	Good % (n)
Women	55.9% (165)	58.7% (91)	52.9% (74)
Men	42.7% (126)	38.7% (61)	46.4% (65)
Non-binary	1.0% (3)	1.3 (2)	0.7% (1)
Demiguy	0.3% (1)	0.6% (1)	0% (0)
**Total**	**100% (295)**	**52.5% (155)**	**47.5% (140)**

Highest Education			
Australian High School/Secondary Education	13.2% (39)	14.2% (22)	12.1% (17)
Cert I-IV/UK High School Diploma/A-levels	17.9% (53)	17.4% (27)	18.6% (26)
Diploma/Advanced Diploma/ Technical/Community College	7.4% (22)	8.4% (13)	6.4% (9)
Bachelor’s Degree/Undergraduate Degree (BA/BSc/Other)	39.9% (118)	39.4% (61)	40.7% (57)
Graduate Diploma/Graduate Certificate/ Graduate Degree (MA/MSc/MPhil/Other)	15.5% (46)	16.8% (26)	14.3% (20)
Postgraduate Degree/Doctorate Degree (PhD/Other)	6.1% (18)	4.5% (7)	7.9% (11)
**Total**	**100% (296)**	**52.7% (156)**	**47.3% (140)**

Ethnicity			
White/Caucasian	83.8% (248)	85.9% (134)	81.4% (114)
Asian/Asian British	9.1% (27)	9.0% (14)	9.3% (13)
Mixed/Multiple Ethnic Groups	3.4% (10)	1.9% (3)	5.0% (7)
Black/African/Caribbean/Black British	3.0% (9)	2.6% (4)	3.6% (5)
Other	0.7% (2)	0.6% (1)	0.7% (1)
**Total**	**100% (296)**	**51.6% (156)**	**48.4% (140)**

Children 2 Years or Younger in Household			
Yes	7.4% (22)	7.7% (12)	7.2% (10)
No	92.5% (273)	92.3% (144)	92.8% (129)
**Total**	**100% (295)**	**52.9% (156)**	**47.1% (139)**

Previous Diagnosis of Sleep Disorder			
Yes	3.7% (11)	5.1% (8)	2.1% (3)
No	96.3% (285)	94.9% (148)	97.9% (137)
**Total**	**100% (296)**	**52.7% (156)**	**47.3% (140)**

Country			
Australia	25.0% (75)	20.1% (32)	30.5% (43)
UK	75.0% (225)	79.9% (127)	69.5% (98)
**Total**	**100% (300)**	**53.0% (159)**	**47.0% (141)**

### Associations of Sleep Hygiene Behaviours with Sleep

Hierarchical multiple regression analyses revealed that previous diagnosis of sleep disorder and country of origin significantly predicted 3.4% of variance (adjusted R^2^ = 0.03) in PSQI scores, *F*(2, 293) = 5.11, *p* = .007. When sleep hygiene behaviours were added to the model, an additional 48.5% of variance was explained, *ΔF*(18, 275) = 15.39, *p* < .001. The overall model accounted for a significant 51.9% of variance (adjusted R^2^ = 0.48) in PSQI scores, *F*(20, 275) = 14.81, *p* < .001, a large effect (*f*^2^ = 1.08). The standardized regression weights for each predictor at each step of the regression are displayed in Table 2. Sleep hygiene behaviours are listed in Step 2 in order of the magnitude of their standardized regression weights.

**Table 2:** Unstandardised (B) and standardised (β) regression coefficients, and squared semi-partial correlations (sr^2^) for variables predicting PSQI scores (*N* = 296)

	Step 1			Step 2		
Predictor	*B* [95% CI]	β	*sr*^*2*^	*B* [95% CI]	β	*sr*^*2*^
Previous Diagnosis of Sleep Disorder	2.29 [0.43, 4.15]*	0.14	.02	0.95 [-0.51, 2.40]	0.06	.00
Country of Origin	0.87 [0.06, 1.68]*	0.12	.01	0.26 [-0.40, 0.90]	0.04	.00
Night-time Worry				0.94 [0.57, 1.31]***	0.26	.04
Different TSTs				0.70 [0.36, 1.04]***	0.21	.03
Sleep Meds				1.00 [0.57, 1.42]***	0.21	.04
Daytime Worry				0.64 [0.23, 1.05]**	0.17	.02
Negative States				0.61 [0.21, 1.01]**	0.17	.02
Checking Time				0.46 [0.17, 0.74]**	0.15	.02
Hungry				0.31 [-0.16, 0.79]	0.06	.00
No Sunlight				0.13 [-0.21, 0.48]	0.04	.00
Thirsty				0.12 [-0.35, 0.59]	0.03	.00
Different Bedtimes				0.06 [-0.32, 0.44]	0.02	.00
Other Activities				0.05 [-0.19, 0.28]	0.02	.00
Different Waketimes				0.04 [-0.32, 0.40]	0.01	.00
Uncomfortable				0.04 [-0.34, 0.42]	0.01	.00
No Relax Time				0.03 [-0.29, 0.35]	0.01	.00
Napping				-0.07 [-0.45, 0.32]	-0.02	.00
Unpleasant Conversation				-0.34 [-0.91, 0.24]	-0.05	.00
Poor Ventilation				-0.27 [-0.75, 0.21]	-0.05	.00
Pondering				-0.24 [-0.59, 0.11]	-0.07	.00

Night-time Worry = Worry about falling asleep while in bed. Different TSTs = Different Total Sleep Times each night. Sleep Meds = Sleep medications. Daytime Worry = Worry about sleep during the day. Negative states = Going to bed feeling stressed out or in other negative states. Checking Time = Checking the time during the night. Hungry = Going to bed while hungry. No sunlight = Having no exposure to sunlight or outdoor light during the day. Thirsty = Going to bed while thirsty. Different Bedtimes = Going to bed at different times. Different Waketimes = Waking up at different times. Other Activities = Doing activities other than sleep in bed (e.g., reading a book, using the computer, watching TV, etc.). Uncomfortable = Going to bed on an uncomfortable bed or pillow/s. No Relax Time = Not enough time to relax before bed. Napping = Napping during the day. Unpleasant Conversation = Having an unpleasant conversation. Poor Ventilation = Going to bed in a poorly ventilated environment. Pondering = Pondering unresolved matters before bed

*CI* confidence interval

^*^p < .05; **p < .01; ***p < .001

### Sleep Hygiene Behaviour Factor Analysis

Principal Components Analysis was conducted with Promax rotation applied, as factors were assumed to correlate, which was confirmed after extraction. The determinant value (.036) was much greater than the cut-off point of .00001 indicating that multicollinearity was not a problem. Both measures of sampling adequacy indicated the data was suitable for factor analysis; Kaiser–Meyer–Olkin = .81, and individual item Measures of Sampling Adequacy were all greater than .5 ranging between .62 and .86. Bartlett’s test of sphericity was significant, χ^2^ (153) = 967.23, *p* < 0.001, further supporting the suitability of the data for factor analysis. Six factors with eigenvalues greater than one were initially extracted although factors five and six had eigenvalues which were only marginally greater than one (1.08 and 1.01 respectively). The scree plot suggested that two or four factors may be more suitable and although parallel analysis confirmed that two factors fit the data best, we also considered the four-factor solution because of several thematically unique items (e.g., napping during the day, exposure to sunlight) which did not load well onto either of the factors in the two-factor model. The four-factor solution was ultimately retained as the total variance accounted for in the data was larger (44.51% compared to 31.12%) and item loadings were stronger across items. Final factor loadings are presented in Table 3.

**Table 3:** Promax rotated factor structure of the sleep hygiene behaviours significantly related to sleep

	Loadings			
Sleep Hygiene Behaviour	Factor 1^a^	Factor 2^b^	Factor 3^c^	Factor 4^d^
Different Waketimes	.79			
Different TSTs	.72			
Different Bedtimes	.71			
Other Activities	.47			
Night-time Worry		.72		
Daytime Worry		.61		
Checking Time		.52		
Sleep Meds		.51		
Uncomfortable		.32		
Hungry			.69	
Thirsty			.57	
Sunlight			.48	
Napping			.40	
Poor Ventilation			.45	
Negative States				.61
No Relax Time				.61
Unpleasant Conversation				.56
Pondering				.51

Only highest loadings are shown for each factor

^a^“Routine”

^b^“Perseverative Cognition”

^c^“Physiological”

^d^“Negative Emotionality”

### Sleep Hygiene Perceptions and Interactions with Sleep

Of the 18 sleep hygiene perceptions that were included in separate multiple regression analyses, four accounted for significant variance in PSQI scores at the Bonferroni corrected level (*p* < .003) after controlling for the frequency in which the sleep hygiene behaviour was performed (see Table 4). Perceptions for having different amounts of time spent asleep each night (B = -0.69, 95% CI = [-1.01, -0.37], *p* < .001), different waketimes (B = -0.59, 95% CI = [-0.94, -0.24], *p* < .001), checking the time during the night (B = -0.57, 95% CI = [-0.90, -0.24], *p* < .001), and screen use (B = -0.63, 95% CI = [-0.98, -0.29], *p* < .001) were negatively related to PSQI scores such that the more negative participants’ perceptions of the impact of behaviour were, the higher their PSQI scores.

**Table 4:** Variance explained for each block of the hierarchical multiple regression analyses where sleep hygiene perceptions or the interaction term significantly predicted PSQI scores (*N* = 296)

	Different TSTs			Different Wake Times			Checking Time			Screen Use			No Sunlight		
Block	*R*^2^	*Δ R*^2^	ΔF *p* value	*R*^2^	*Δ R*^2^	ΔF *p* value	*R*^2^	*Δ R*^2^	ΔF *p* value	*R*^2^	*Δ R*^2^	ΔF *p* value	*R*^2^	*Δ R*^2^	ΔF *p* value
Control Variables	.03	.034	.007	.03	.03	.007	.03	.03	.007	.03	.03	.007	.03	.03	.007
Behavioural Performance	.20	.17	< .001	.10	.06	< .001	.15	.11	< .001	.04	.01	.159	.09	.06	< .001
Perception	.25	.05	< .001	.13	.03	< .001	.18	.03	< .001	.08	.04	< .001	.09	.00	.895
Performance X Perception	.25	.00	.611	.13	.00	.241	.20	.02	.005	.08	.00	.623	.12	.03	.003

Control Variables = Previous Diagnosis of Sleep Disorder and Country of Origin. Behavioural Performance = Frequency of respective sleep hygiene behaviour performance over previous week. Perception = Perception of respective sleep hygiene behaviours. Performance X Perception = Interaction term between behavioural performance and perception

**Fig. 2:**
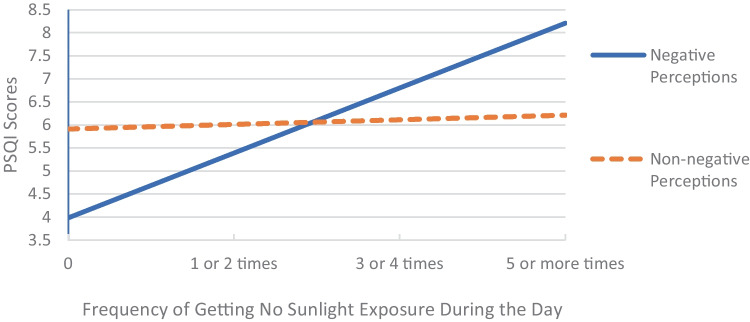
Association Between No Sunlight Exposure and Sleep Moderated by Perceptions

**Fig. 3:**
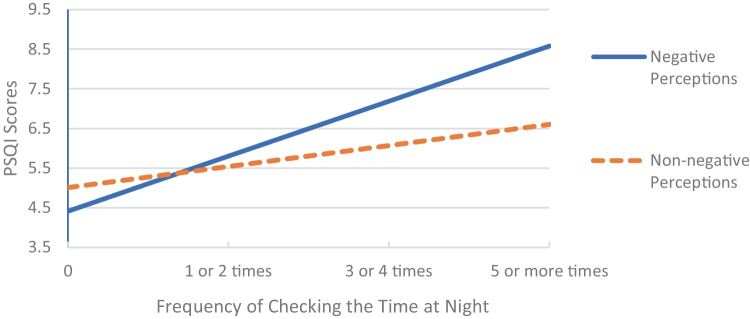
Association Between Checking the Time at Night and Sleep Moderated by Perceptions

Not getting exposure to sunlight or outdoor light during the day demonstrated a significant interaction (*p* = .003) between sleep hygiene perception, sleep hygiene behaviour performance and sleep, such that the association of getting no sunlight during the day with sleep was only significant for participants with negative perceptions (B = 1.40, 95% CI = [0.92, 1.87], *p* < .001), i.e., those that believed it impaired their sleep (Fig. 2). Another interaction was observed for checking the time during the night, whereby the association of checking the time with sleep was more pronounced for participants with negative perceptions. After Bonferroni corrections the overall interaction was not significant (*p* = .005), however the interaction is still presented in Fig. 3 to demonstrate the direction.

## Discussion

The results from this study show the degree of association between a broad range of sleep hygiene behaviours and sleep quality. Of the 35 behaviours which were included, only 18 were significantly associated with sleep quality. The predictors which had the biggest association with sleep (ranked in order based on the magnitude of their standardized regression coefficients) were; worrying about falling asleep while in bed, spending different amounts of time each night sleeping, using sleep medications, worrying about sleep during the day, feeling stressed out or being in other negative states before bed, checking the time in the middle of the night, going to bed hungry, getting no exposure to sunlight or outdoor light during the day, going to bed thirsty, going to bed at different times, using the bed for activities other than sleep, going to bed on an uncomfortable bed or pillow/s, waking up at different times, not having enough time to relax before bed, napping during the day, having an unpleasant conversation before bed, going to bed in an environment that is poorly ventilated, and pondering unresolved matters before bed. Conversely, falling asleep with the TV or music on, exercise (neither vigorous or moderate intensity), caffeine or other stimulating substance use, alcohol use, eating or drinking too much before bed, having sleep interrupted by a partner or pets, going to sleep in environments that were too noisy (or too quiet), too bright (or too dark), or too humid (or too dry), engaging in activities of high concentration before bed, exposure to blue light before bed, and using alcohol with the explicit intention of helping to fall asleep, were not significantly related to sleep.

Of the significant behaviours, only the first six explained unique variance in sleep, which indicated that there was considerable shared variance between the other variables. One potential reason for this is the overlap in content between some of these behaviours. For example, it is entirely plausible that worrying about sleep during the day influences sleep through the same mechanism that worrying about sleep during the night does. Perseverative cognition, which includes repetitive negative thoughts such as worry or rumination has been demonstrated to negatively impact sleep [27, 28] and by this definition, it serves as a plausible underlying mechanism by which the aforementioned sleep hygiene behaviours affect sleep. Likewise, frequent checking of the time in the middle of the night can increase worry about meeting sleep needs [29]. This is reinforced by the results of the factor analysis which showed that these worry-type behaviours (daytime worry, night-time worry, and checking the time in the middle of the night) loaded onto one factor. Use of sleep medication also loaded onto this factor, but ruminative patterns of thinking such as perseverative cognition are strongly associated with depression and anxiety [30, 31], which in turn are predictors of sleep medication [32]. Therefore, it was not surprising to see sleep medications load onto this factor, given the intercorrelation.

Similarly, some of the other variables like pondering unresolved matters before bed or feeling stressed out could conceivably operate under a shared mechanism too. Research has shown that negative emotionality is associated with greater pre-sleep arousal [33]. Therefore, it is possible that the sleep hygiene behaviours related to emotional arousal in the present study (e.g., having an unpleasant conversation before bed, feeling stressed out, not having enough time to relax) affect sleep through this common mechanism. This was reflected by the results of the factor analysis which showed that they loaded onto the same factor.

The results also indicated the importance of routine in sleep quality, as all three routine-based variables were associated with sleep. Going to bed and waking up at different times, and therefore having a consistent length of time spent asleep each night, has been demonstrated to be important in maintaining good quality sleep. For example, research highlighting higher levels of sleep difficulties among shift workers demonstrate the effects variable sleep schedules can have on sleep compared to their non-variable counterparts [34, 35]. Factor analysis also showed these variables grouping together; thus, the present findings reinforce the inclusion of these variables as important sleep hygiene behaviours. Additionally, using the bed for activities other than sleep loaded on this factor too, which aligns with findings that suggest adult bedtime activities occur consistently and without much thought [36]. This might reflect the performance of other activities in bed like reading, checking emails, or using social media in bed as being habitually performed [37] and therefore belonging to a general routine factor alongside setting a consistent sleep schedule.

Other sleep hygiene behaviours which did not account for unique variance in sleep, but which were independently associated with sleep seemed to share variance in the outcome variable and were also reflected in the factor analysis by loading onto a single factor which we categorised as a physiological factor. These five sleep hygiene behaviours included going to bed hungry or thirsty, napping during the day, not getting exposure to sunlight or outdoor light during the day, and going to bed in a poorly ventilated environment. Although each of these sleep hygiene behaviours are proposed to effect sleep in distinctly different ways [38–41], they share a commonality in that they are all involved in a physiological process. For example, poor ventilation can reduce oxygen supply [38], hunger and thirst are markers that signal a survival need [42], napping is often a response to a deficiency in sleep needs [41], and sunlight helps entrain the circadian rhythm by regulating melatonin production [39]. As such, the common variance in these items could represent a grouping of behaviours which contribute to sleep through biological or physiological means.

Use of sleep medication was positively associated with PSQI scores which indicate that more frequent use was related to poorer sleep. This initially seems counterintuitive, since sleep medications ought to improve sleep, not impair it. However, this association has been demonstrated in previous literature [43]. A potential explanation for this is that those that use sleep medication are often inherently poorer sleepers who turn to sleep medications, which may not always improve their subjective sleep quality. Furthermore, prolonged use can lead to altered sleep physiology, and built-up tolerance effects may render their ability to improve sleep minimal [44]. Therefore, the observed negative association may reflect poorer sleepers turning to sleep medications, rather than a causative effect of sleep medications decreasing sleep quality.

While there is much to be said about those behaviours that were significantly related to sleep, attention should also be given to those that were not. For some behaviours, the lack of association with sleep may be explainable by properties of the behaviour itself. That is, certain thresholds or conditions of performance of the behaviour may be more important to consider than whether there is an overall effect (e.g., the quantity of alcohol, or the timing of exercise before bed). To illustrate further, consider the impact of exposure to blue light on sleep as an example, a behaviour which was not significantly related to sleep in the present study.

Although research has demonstrated that blue light can interrupt sleep patterns by suppressing melatonin [45], blue blocking lenses have been shown to have limited efficacy in improving sleep quality [46]. Furthermore, other studies have compared blue light blocking filters on smartphones and tablets compared to unfiltered screens and found no differences in sleep outcomes and melatonin suppression (e.g., [47]). However, these studies did not test these effects at exposure times greater than two hours, and since the item in the present study did not ask participants to quantify the source of blue light nor the amount of time exposed, it is possible that effects of blue light may only significantly impact sleep under certain parameters (i.e., long exposure times). This effect has been demonstrated by Heo et al. [48] who assessed exposure at 150 min to observe a significant effect on sleepiness, and may explain the success of blue-light blocking lens during the evening at workplaces [49], where it is presumed that blue-light exposure at workplace is also greater than two hours. The subjective recall of the behaviour in the present study might also have contributed to the null finding if this effect is indeed true.

A critical lens applied to the remaining non-significant behaviours might elicit other plausible explanations for each behaviour, although the required level of detail to achieve this goes beyond the scope of the present research and might be best suited in a narrative review. Instead, we reiterate that many of the behaviours which were not related to sleep may be better explained by specific characteristics of the behaviour itself (e.g., time of performance in relation to sleep, intensity of the behaviour), and deserve appropriate attention to disentangle thresholds or conditions in which they impact sleep. Although not all of these specific characteristics of behaviour were explored in the present study, the results do suggest that one such characteristic (individual perceptions) may serve as an additional factor to consider when exploring the relationship between sleep hygiene behaviours and sleep.

Four perceptions (checking the time during the night, screen use before bed, waking up at different times each day, and spending different lengths of time asleep each night) were significantly associated with sleep over and above simply performing the respective behaviour. This indicates that in addition to performing a detrimental sleep hygiene behaviour, believing that this performance was impairing sleep was able to explain additional variance in some of the behaviours examined.

An obvious interpretation of these findings is that having strong negative beliefs about the impact of a behaviour is simply reflective of an inherent underlying tendency to worry or ruminate which in turn is associated with poorer sleep [50]. While this might be the case for some associations observed, the interaction effects demonstrated in the present study suggest that the role of perceptions may go further than this. For two behaviours (not getting enough exposure to sunlight/outdoor light and checking the time during the night) the results show that the associations of performing the behaviour with sleep were moderated by negative perceptions about the personal impact of the behaviour. For checking the time, the relationship was exaggerated by negative perceptions, and for no sunlight exposure, the relationship was only present when perceptions were negative. These findings are novel and although they were not replicated for each behaviour examined in the present population, they do suggest that the role of perceptions about sleep hygiene behaviours on sleep ought to be examined further (e.g., whether controlling negative perceptions of sleep hygiene behaviours leads to better sleep).

### Implications

The present research findings have public health implications through several avenues. Exploratory factor analyses importantly revealed the clustering of several behaviours that were drawn across three commonly used sleep hygiene scales. Researchers are therefore able to consider choosing one or more domains that tailor to their specific needs. For instance, schedule-type behaviours may be of particular importance for research with shift workers, whereas behaviours that fall under perseverative cognition could be applied to research in populations that are more likely to reflect this psychopathology. Furthermore, the use of distinct domains may also provide insight for GPs and clinicians into specific problematic areas and allow for a more targeted approach when developing treatment plans. Rather than treating poor sleep hygiene as an overall deficit, attempts to target a specific domain with related problematic behaviours may yield more efficacious results while reducing the demand on resources for both patient and clinician.

### Strengths, Limitations, and Future Directions

The present exploratory examination of accepted sleep hygiene behaviours provided more support for the inclusion of some behaviours. However, one limitation of the present research was the use of subjective measures to account for sleep outcomes. Differences between subjective and objective measures have been demonstrated previously [51] and a combination of both would be useful to strengthen the certainty from which conclusions can be made. Similarly, the cross-sectional design limits the extent to which confounding variables could be accounted for. Experimental studies which manipulate the exposure to different sleep hygiene variables will be important to determine the causal impact of individual behaviours on sleep. However, the use of a cross-sectional design allowed for the unobtrusive examination of the much more realistic possibility where multiple sleep hygiene behaviours are performed each night, allowing for valuable inferences for many behaviours to be drawn simultaneously from a naturalistic setting. Future research could however look to adopt repeated measures designs that assess day-to-day fluctuations in sleep outcomes based on variations in the presence/absence of specific sleep hygiene behaviours, particularly for those behaviours which did not relate significantly to sleep. Adopting such methodologies which draw on the present findings, could progress knowledge within the field to the point where a new sleep hygiene scale could be developed.

There are also some potential limitations associated with recruitment via paid online participant pools [52], the most commonly cited are the potential for limited attention and higher risk of biased responding (e.g., by demand characteristics of the researcher). However, the present study included three measures to combat such concerns including use of attention checks, recruitment of only participants with high (> 95%) approval ratings, and recruitment of only participants who had completed more than 100 studies. When implemented, such measures have been shown to produce high quality data in Prolific, and thus we considered the sample to be a relatively valid representation of a broad cross-section of the general population [53]. However, given the recruitment method, the findings may not be generalisable to other more specific samples of interest (e.g., shift workers or disordered populations).

In combination, the results provide several directions for future research. Firstly, additional evidence has been garnered for several sleep hygiene behaviours, which ought to serve as a basis for which behaviours to include for future sleep hygiene scale development in general populations. Concurrently, the findings also highlight many sleep hygiene behaviours which were not associated with sleep problems in the general population, flagging areas which ought to be examined more critically (e.g., by further exploring the conditions in which a behaviour *is* related to sleep) before being considered for inclusion in the refinement and validation of a sleep hygiene scale for non-clinical populations. Alternatively, techniques for improving sleep in non-clinical populations may benefit from focusing on a smaller number of self-selected behaviours [54], or by targeting discrete sleep hygiene factors, rather than focusing on numerous individual behaviours within existing scales. This conclusion is supported by the limited effect of non-significant behaviours demonstrated here and in other research, the practical difficulty associated with having to control many behaviours each day to enhance sleep, and the results from the factor analysis which suggest that many behaviours share an underlying mechanism anyway.

## Conclusion

Overall, the research further highlights the complexity of the relationship between sleep hygiene behaviours and sleep and draws attention to the need to assess sleep hygiene behaviours more critically. Routine-based factors such as going to bed and waking up at the same time and spending the same amount of time sleeping each night were important predictors of sleep. Behaviours that reflected perseverative cognitions (worrying about sleep during the day or at night and checking the time in the middle of the night) were also related to sleep, as were behaviours that elicit emotional arousal (being in a stressed or negative emotional state, having an unpleasant conversation, and not having enough time to relax before bed). Other behaviours that should be considered as negatively associated with sleep include not getting exposure to sunlight during the day, going to bed hungry or thirsty, using the bed for activities other than sleep, going to bed in a poorly ventilated environment, and sleeping on an uncomfortable mattress and/or pillows. Non-significant findings for approximately half of the most frequently used sleep hygiene behaviours warrants a closer inspection as to the situational and individual/dispositional characteristics that may influence whether these behaviours impact sleep (if at all). However, future research in the area should also consider narrowing the focus to shared common mechanisms, as well as incorporating perceptions of sleep hygiene to further establish the role they play in sleep.

## Supplementary Information

Below is the link to the electronic supplementary material.

Supplementary file1 (DOCX 23 KB)

## Data Availability

The data underlying this article cannot be shared publicly due to the conditions under which ethics were obtained. Participants were informed that only “The following people will have access to the information we collect in this research: the research team and, in the event of an audit or investigation, staff from the Curtin University Office of Research and Development”. As such we are unable to make data publicly available.

## Footnotes

1 For more information about data screening and how the assumption violations were handled please see supporting information (Supplementary 2).

## References

[1] Hillman DR, Lack LC. Public health implications of sleep loss: The community burden. Med J Aust. 2013;199:S7–10. 10.5694/mja13.10620. 24138358 10.5694/mja13.10620

[2] Liu Y, Wheaton AG, Chapman DP, Croft JB. Sleep duration and chronic diseases among US adults age 45 years and older: Evidence from the 2010 Behavioral Risk Factor Surveillance System. Sleep. 2013;36(10):1421–7. 10.5665/sleep.3028. 24082301 10.5665/sleep.3028 PMC3773191

[3] Geiger-Brown J, Rogers VE, Trinkoff AM, Kane RL, Bausell RB, Scharf SM. Sleep, sleepiness, fatigue, and performance of 12-hour-shift nurses. Chronobiol Int. 2012;29(2):211–9. 10.3109/07420528.2011.645752. 22324559 10.3109/07420528.2011.645752

[4] Kazem YM, El Shebini SM, Moaty MI, Fouad S, Tapozada ST. Sleep deficiency is a modifiable risk factor for obesity and cognitive impairment and associated with elevated visfatin. Open Access Macedonian J Med Sci. 2015;3(2):315. 10.3889/oamjms.2015.063. 10.3889/oamjms.2015.063 PMC4877875 27275243

[5] Rangaraj VR, Knutson KL. Association between sleep deficiency and cardiometabolic disease: Implications for health disparities. Sleep Med. 2016;18:19–35. 10.1016/j.sleep.2015.02.535. 26431758 10.1016/j.sleep.2015.02.535 PMC4758899

[6] Sutton EL. Psychiatric disorders and sleep issues. Med Clin. 2014;98(5):1123–43. 10.1016/j.mcna.2014.06.009 25134876

[7] Hafner M, Stepanek M, Taylor J, Troxel WM, Van Stolk C. Why sleep matters—the economic costs of insufficient sleep: a cross-country comparative analysis. Rand Health Quar 2017;6(4). 10.7249/rb9962. 10.7249/rhq-06-04-11 PMC5627640 28983434

[8] Li J, Zhou K, Li X, et al. Mediator effect of sleep hygiene practices on relationships between sleep quality and other sleep-related factors in Chinese mainland university students. Behav Sleep Med. 2016;14(1):85–99. 10.1080/15402002.2014.954116. 25356919 10.1080/15402002.2014.954116

[9] Knufinke M, Nieuwenhuys A, Geurts SAE, Coenen AML, Kompier MAJ. Self-reported sleep quantity, quality and sleep hygiene in elite athletes. J Sleep Res. 2018;27(1):78–85. 10.1111/jsr.12509. 28271579 10.1111/jsr.12509

[10] Ali RM, Zolezzi M, Awaisu A. A systematic review of instruments for the assessment of insomnia in adults. Nat Sci Sleep. 2020;12:377–409. 10.2147/NSS.S250918. 32753991 10.2147/NSS.S250918 PMC7342485

[11] Jefferson CD, Drake CL, Scofield HM, et al. Sleep hygiene practices in a population-based sample of insomniacs. Sleep. 2005;28(5):611–5. 10.1093/sleep/28.5.611. 16171275 10.1093/sleep/28.5.611

[12] Cheek RE, Shaver JLF, Lentz MJ. Variations in sleep hygiene practices of women with and without insomnia. Res Nurs Health. 2004;27(4):225–36. 10.1002/nur.20025. 15264262 10.1002/nur.20025

[13] Stepanski EJ, Wyatt JK. Use of sleep hygiene in the treatment of insomnia. Sleep Med Rev. 2003;7(3):215–25. 10.1053/smrv.2001.0246. 12927121 10.1053/smrv.2001.0246

[14] Mastin DF, Bryson J, Corwyn R. Assessment of sleep hygiene using the Sleep Hygiene Index. J Behav Med. 2006;29(3):223–7. 10.1007/s10865-006-9047-6. 16557353 10.1007/s10865-006-9047-6

[15] Lacks P, Rotert M. Knowledge and practice of sleep hygiene techniques in insomniacs and good sleepers. Behav Res Ther. 1986;24(3):365–8. 10.1016/0005-7967(86)90197-x. 3729908 10.1016/0005-7967(86)90197-x

[16] Yang C-M, Lin S-C, Hsu S-C, Cheng C-P. Maladaptive sleep hygiene practices in good sleepers and patients with insomnia. J Health Psychol. 2010;15(1):147–55. 10.1177/1359105309346342. 20064894 10.1177/1359105309346342

[17] Bootzin RR. Stimulus control treatment for insomnia. Proc Am Psychol Assoc. 1972;7:395–6. 10.1037/e465522008-198.

[18] Gellis LA, Lichstein KL. Sleep hygiene practices of good and poor sleepers in the United States: An internet-based study. Behav Ther. 2009;40(1):1–9. 10.1016/j.beth.2008.02.001. 19187812 10.1016/j.beth.2008.02.001

[19] Otte JL, Wu J, Yu M, Shaw C, Carpenter JS. Evaluating the Sleep Hygiene Awareness and Practice Scale in midlife women with and without breast cancer. J Nurs Meas. 2016;24(2):258–67. 10.1891/1061-3749.24.2.258. 27535313 10.1891/1061-3749.24.2.258 PMC13087921

[20] Voinescu BI, Szentagotai-Tatar A. Sleep hygiene awareness: its relation to sleep quality and diurnal preference. J Mol Psychiatry. 2015;3(1):1–7. 10.1186/s40303-015-0008-2. 25810915 10.1186/s40303-015-0008-2 PMC4328962

[21] Palagini L, Ong JC, Riemann D. The mediating role of sleep-related metacognitive processes in trait and pre-sleep state hyperarousal in insomnia disorder. J Psychosom Res. 2017;99:59–65. 10.1016/j.jpsychores.2017.03.001. 28712431 10.1016/j.jpsychores.2017.03.001

[22] Morin CM, Vallières A, Ivers H. Dysfunctional Beliefs and Attitudes about Sleep (DBAS): Validation of a Brief Version (DBAS-16). Sleep. 2007;30(11):1547–54. 10.1093/sleep/30.11.1547. 18041487 10.1093/sleep/30.11.1547 PMC2082102

[23] Buysse DJ, Reynolds CF, Monk TH, Berman SR, Kupfer DJ. The Pittsburgh sleep quality index: A new instrument for psychiatric practice and research. Psychiatry Res. 1989;28(2):193–213. 10.1016/0165-1781(89)90047-4. 2748771 10.1016/0165-1781(89)90047-4

[24] Carson V, Adamo K, Rhodes RE. Associations of parenthood with physical activity, sedentary behavior, and sleep. Am J Health Behav. 2018;42(3):80–9. 10.5993/AJHB.42.3.8. 29663983 10.5993/AJHB.42.3.8

[25] Allen P, Bennett K, Heritage B. SPSS statistics version 22: A practical guide. 3 edn: Cengage Learning Australia; 2014.

[26] Aguinis H, Gottfredson RK. Best-practice recommendations for estimating interaction effects using moderated multiple regression. J Organ Behav. 2010;31(6):776–86. 10.1002/job.686.

[27] Clancy F, Prestwich A, Caperon L, Tsipa A, O’Connor DB. The association between worry and rumination with sleep in non-clinical populations: a systematic review and meta-analysis. Health Psychol Rev. 2020;14(4):427–48. 10.1080/17437199.2019.1700819. 31910749 10.1080/17437199.2019.1700819

[28] Tutek J, Gunn HE, Lichstein KL. Worry and rumination have distinct associations with nighttime versus daytime sleep symptomology. Behav Sleep Med. 2021;19(2):192–207. 10.1080/15402002.2020.1725012. 32036690 10.1080/15402002.2020.1725012

[29] Riemann D, Fischer J, Mayer G, Peter HJ. The guidelines for ‘non-restorative sleep’: Relevance for the diagnosis and therapy of insomnia. Somnologie-Schlafforschung und Schlafmedizin. 2003;7(2):66–76. 10.1046/j.1439-054x.2003.03201.x.

[30] Ottaviani C, Shahabi L, Tarvainen M, Cook I, Abrams M, Shapiro D. Cognitive, behavioral, and autonomic correlates of mind wandering and perseverative cognition in major depression. Front Neurosci. 2015;8:433. 10.3389/fnins.2014.00433. 10.3389/fnins.2014.00433 PMC4283544 25601824

[31] Sorg S, Vögele C, Furka N, Meyer A. Perseverative thinking in depression and anxiety. Front Psychol. 2012;3:20. 10.3389/fpsyg.2012.00020. 10.3389/fpsyg.2012.00020 PMC3277932 22347869

[32] Omvik S, Pallesen S, Bjorvatn B, Sivertsen B, Havik OE, Nordhus IH. Patient characteristics and predictors of sleep medication use. Int Clin Psychopharmacol. 2010;25(2):91–100. 10.1097/YIC.0b013e328334e5e6. 20071997 10.1097/YIC.0b013e328334e5e6

[33] Hantsoo L, Khou CS, White CN, Ong JC. Gender and cognitive–emotional factors as predictors of pre-sleep arousal and trait hyperarousal in insomnia. J Psychosom Res. 2013;74(4):283–9. 10.1016/j.jpsychores.2013.01.014. 23497828 10.1016/j.jpsychores.2013.01.014 PMC3655522

[34] Thach T-Q, Mahirah D, Dunleavy G, et al. Association between shift work and poor sleep quality in an Asian multi-ethnic working population: A cross-sectional study. PLoS One. 2020;15(3):e0229693. 10.1371/journal.pone.0229693. 10.1371/journal.pone.0229693 PMC7055880 32130268

[35] Matsangas P, Shattuck NL, Saitzyk A. Sleep-related practices, behaviors, and sleep-related difficulties in deployed active-duty service members performing security duties. Behav Sleep Med. 2020;18(2):262–74. 10.1080/15402002.2019.1578771. 30764663 10.1080/15402002.2019.1578771

[36] Koketsu J. Pre-sleep routines in adult normal sleepers. Eastern Kentucky University; 2018.

[37] Exelmans L, Scott H. Social media use and sleep quality among adults: The role of gender, age and social media checking habit. 2019. 10.31234/osf.io/eqxdh.

[38] Strøm-Tejsen P, Zukowska D, Wargocki P, Wyon DP. The effects of bedroom air quality on sleep and next-day performance. Indoor Air. 2016;26(5):679–86. 10.1111/ina.12254. 26452168 10.1111/ina.12254

[39] Roenneberg T, Kantermann T, Juda M, Vetter C, Allebrandt KV. Light and the human circadian clock. In: Kramer A, Merrow M, editors. Circadian clocks. Berlin, Heidelberg: Springer Berlin Heidelberg; 2013. p. 311–31. 10.1007/978-3-642-25950-0_13 23604485

[40] Nicolaidis S. Metabolic mechanism of wakefulness (and hunger) and sleep (and satiety): Role of adenosine triphosphate and hypocretin and other peptides. Metabolism. 2006;55:S24–9. 10.1016/j.metabol.2006.07.009. 16979423 10.1016/j.metabol.2006.07.009

[41] Dhand R, Sohal H. Good sleep, bad sleep! The role of daytime naps in healthy adults. Curr Opin Pulm Med. 2006;12(6):379–82. 10.1097/01.mcp.0000245703.92311.d0. 17053484 10.1097/01.mcp.0000245703.92311.d0

[42] Sternson SM. Hypothalamic survival circuits: Blueprints for purposive behaviors. Neuron. 2013;77(5):810–24. 10.1016/j.neuron.2013.02.018. 23473313 10.1016/j.neuron.2013.02.018 PMC4306350

[43] Choi NG, DiNitto DM, Marti CN, Choi BY. Too little sleep and too much sleep among older adults: Associations with self-reported sleep medication use, sleep quality and healthcare utilization. Geriatr Gerontol Int. 2017;17(4):545–53. 10.1111/ggi.12749. 27195448 10.1111/ggi.12749

[44] Kripke DF. Chronic hypnotic use: Deadly risks, doubtful benefit: Review article. Sleep Med Rev. 2000;4(1):5–20. 10.1053/smrv.1999.0076. 12531158 10.1053/smrv.1999.0076

[45] West KE, Jablonski MR, Warfield B, et al. Blue light from light-emitting diodes elicits a dose-dependent suppression of melatonin in humans. J Appl Physiol. 2010;110(3):619–26. 10.1152/japplphysiol.01413.2009. 21164152 10.1152/japplphysiol.01413.2009

[46] Janků K, Šmotek M, Fárková E, Kopřivová J. Block the light and sleep well: Evening blue light filtration as a part of cognitive behavioral therapy for insomnia. Chronobiol Int. 2020;37(2):248–59. 10.1080/07420528.2019.1692859. 31752544 10.1080/07420528.2019.1692859

[47] Heath M, Sutherland C, Bartel K, et al. Does one hour of bright or short-wavelength filtered tablet screenlight have a meaningful effect on adolescents’ pre-bedtime alertness, sleep, and daytime functioning? Chronobiol Int. 2014;31(4):496–505. 10.3109/07420528.2013.872121. 24397302 10.3109/07420528.2013.872121

[48] Heo J-Y, Kim K, Fava M, et al. Effects of smartphone use with and without blue light at night in healthy adults: A randomized, double-blind, cross-over, placebo-controlled comparison. J Psychiatr Res. 2017;87:61–70. 10.1016/j.jpsychires.2016.12.010. 28017916 10.1016/j.jpsychires.2016.12.010

[49] Guarana CL, Barnes CM, Ong WJ. The effects of blue-light filtration on sleep and work outcomes. J Appl Psychol. 2020;106(5):784–96. 10.1037/apl0000806. 32658494 10.1037/apl0000806

[50] Slavish DC, Graham-Engeland JE. Rumination mediates the relationships between depressed mood and both sleep quality and self-reported health in young adults. J Behav Med. 2015;38(2):204–13. 10.1007/s10865-014-9595-0. 25195078 10.1007/s10865-014-9595-0 PMC4362854

[51] Jackowska M, Dockray S, Hendrickx H, Steptoe A. Psychosocial factors and sleep efficiency: Discrepancies between subjective and objective evaluations of sleep. Psychosom Med. 2011;73(9):810–6. 10.1097/PSY.0b013e3182359e77. 22021463 10.1097/PSY.0b013e3182359e77

[52] Palan S, Schitter C. Prolific.ac — A subject pool for online experiments. J Behav Experiment Fin. 2018;17:22–7. 10.1016/j.jbef.2017.12.004.

[53] Peer E, Rothschild D, Gordon A, Evernden Z, Damer E. Data quality of platforms and panels for online behavioral research. 54.4(1554–3528 (Electronic)):1643–62. 10.3758/s13428-021-01694-3. 10.3758/s13428-021-01694-3 PMC8480459 34590289

[54] Kor K, Mullan BA. Sleep hygiene behaviours: An application of the theory of planned behaviour and the investigation of perceived autonomy support, past behaviour and response inhibition. Psychol Health. 2011;26(9):1208–24. 10.1080/08870446.2010.551210. 21678170 10.1080/08870446.2010.551210

