# Supplementary material for: Re-considering the Role of Sleep Hygiene Behaviours in Sleep: Associations Between Sleep Hygiene, Perceptions and Sleep
Source: Int J Behav Med. 2023 Sep 6;31(5):705–17. doi: 10.1007/s12529-023-10212-y (PMC11452418; doi:10.1007/s12529-023-10212-y)
Supplement: Supplementary file 1 — Supplementary file1 (DOCX 23 KB) [file 12529_2023_10212_MOESM1_ESM.docx]

**Supplementary Material 1**

| Sleep Hygiene Behaviour |  | Pearson’s r |  | p-value |
| --- | --- | --- | --- | --- |
| **Worrying about falling asleep while in bed** |  | **.53***** |  | **< .001** |
| **Having an unpleasant conversation before bed** |  | **.15*** |  | **.012** |
| **Not having enough time to relax before bed** |  | **.14*** |  | **.017** |
| Falling asleep with music or the TV on |  | .09 |  | .107 |
| **Checking the time in the middle of the night** |  | **.36***** |  | **< .001** |
| **Worrying about sleep during the day** |  | **.49***** |  | **< .001** |
| **Using the bed for activities other than sleep** |  | **.14*** |  | **.020** |
| **Going to bed at different times** |  | **.27***** |  | **< .001** |
| **Waking up at different times** |  | **.27***** |  | **< .001** |
| **Spending different amounts of time each night sleeping** |  | **.41***** |  | **< .001** |
| Exercising vigorously before bed |  | -.07 |  | .202 |
| Engaging in moderate intensity exercise before bed |  | -.05 |  | ..394 |
| **Going to bed hungry** |  | **.25***** |  | **< .001** |
| **Going to bed thirsty** |  | **.22***** |  | **< .001** |
| Drinking too much water before bed |  | .11 |  | .065 |
| Eating too much before bed |  | .07 |  | .240 |
| **Napping during the day** |  | **.14*** |  | **.014** |
| **Pondering unresolved matters before bed** |  | **.27***** |  | **< .001** |
| Having your sleep interrupted by pets |  | .10 |  | .077 |
| Having sleep interrupted by a partner |  | .06 |  | .317 |
| **Having no exposure to sunlight or outdoor light during the day** |  | **.26***** |  | **< .001** |
| Using a screen (phone, computer, laptop, TV, etc.) immediately before bed |  | .08 |  | .176 |
| Going to sleep in an environment that is too noisy (or quiet) |  | .11 |  | .051 |
| Going to sleep in an environment that is too bright (or dark) |  | .04 |  | .528 |
| Going to sleep in an environment that is too humid (or dry) |  | .08 |  | .174 |
| **Going to sleep in an environment that is poorly ventilated** |  | **.13*** |  | **.027** |
| **Going to sleep on an uncomfortable bed or pillow/s** |  | **.18**** |  | **.001** |
| Having caffeine before bed |  | .07 |  | .212 |
| Consuming any other stimulating substances before bed (e.g., Nicotine) |  | .00 |  | .971 |
| Engaging in activities requiring high concentration before bed |  | .07 |  | .21 |
| Drinking alcohol before bed |  | -.05 |  | .397 |
| Drinking alcohol with the intention of using it to fall asleep |  | .09 |  | .121 |
| **Using sleep medications to help sleep** |  | **.32***** |  | **< .001** |
| **Feeling stressed out or in another negative state** |  | **.41***** |  | **< .001** |
| Being exposed to blue light before bed (blue light can be emitted from sources like your laptop if they don’t have ‘night light’ enabled, or LED or fluorescent lighting) |  | .04 |  | .507 |

Pearson Correlation Coefficients for all Sleep Hygiene Behaviours with Sleep

Note. *p < .05, **p < .01, ***p < .001

**Supplementary Material 2**

Data Screening and Analyses Information

Fourteen participants reported sleep efficiencies over 100%. These data were corrected to 100%. A further 13 participants missed or mislabelled their bedtime or waketime as the wrong meridiem code (e.g., no AM or PM was reported), and these were adjusted and retained if only one option made practical sense, otherwise, data was left as missing. A total of 12 cases were missing on the age variable, 5 from gender, 5 from children two years and younger living in the household, 4 each from education, ethnicity, and previous diagnosis of sleep disorder, and 22 were missing the PSQI other difficulties item. These were checked and for every case, missingness was ignored and the total PSQI score was used as it was, because any possible answer that may have been given by the participant in each case was unable to affect the total PSQI score (outcome variable). The remaining 12 cases had their ages imputed using expectation maximisation, whereas gender and the other categorical control/demographic variables could not be imputed so data was left as missing.

The assumptions of multiple regression were tested with minor violations occurring. Several variables displayed positive skews which reflected the limited engagement of the population sample in some of the sleep hygiene variables during the past week. However, Knief and Forstmeier (2021) argue that for multiple regression, normality is not hugely critical. No variables had univariate outliers greater than three standard deviations away from the mean. Twelve multivariate outliers were identified at the critical chi-square value for 20 predictors (χ^2^ = 45.315) but their influence as indicated by Cook’s value was negligible (Allen et al., 2014). The remaining assumptions were met.

**References**

Allen, P., Bennett, K., & Heritage, B. (2014). *SPSS statistics version 22: A practical guide* (3 ed.). Cengage Learning Australia.

Knief, U., & Forstmeier, W. (2021). Violating the normality assumption may be the lesser of two evils. *Behavior Research Methods*, 1-15.
